# Physiological Responses of a Diazotrophic Cyanobacterium to Acidification of Paddy Floodwater: N_2_ Fixation, Photosynthesis, and Oxidative–Antioxidative Characteristics

**DOI:** 10.3390/ijerph192215070

**Published:** 2022-11-16

**Authors:** Qiong Yan, Peng Xiao, Jun Li, Yaxian He, Jihai Shao

**Affiliations:** 1 College of Resources and Environment, Hunan Agricultural University, Changsha 410128, China; 2 National and Local Joint Engineering Research Center of Ecological Treatment Technology for Urban Water Pollution, College of Life and Environmental Science, Wenzhou University, Wenzhou 325035, China; 3 National Engineering Research Center for Agrochemicals/Hunan Provincial Key Laboratory of Agrochemicals, Hunan Research Institute of Chemical Industry, Changsha 410014, China; 4 Zhuzhou Ecology and Environment Monitoring Center, Zhuzhou 412000, China

**Keywords:** acidification, diazotrophic cyanobacteria, photosynthesis, reactive oxygen species, antioxidants

## Abstract

Long-term of excessive fertilization using nitrogen (N) chemical fertilizer caused the acidification of paddy soils. Presently, the impacts of soil acidification on physiological characteristics of diazotrophic cyanobacteria remain unknown. In order to elucidate this issue, the effects of paddy floodwater acidification on activities of respiration, photosynthetic oxygen evolution, and N_2_ fixation of a paddy diazotrophic cyanobacterium *Aliinostoc* sp. YYLX235 were investigated in this study. In addition, the origination and quenching of intracellular reactive oxygen species (ROS) were analyzed. The acidification of paddy floodwater decreased intracellular pH and interfered in energy flux from light-harvesting chlorophyll antenna to the reaction center of photosystem II (PS II). Activities of respiration, photosynthetic oxygen evolution, and N_2_ fixation were decreased by the acidification of paddy floodwater. Accompanied with an increase in ROS, the level of antioxidative system increased. Superoxide dismutase (SOD) and catalase (CAT) were the main enzymatic ROS scavengers in the cells of YYLX235; reduced glutathione (GSH) was the main non-enzymatic antioxidant. Antioxidants and oxidants in the cells of YYLX235 lost balance when the pH of paddy floodwater fell to 5.0 and 4.0, and lipid oxidative damage happened. The results presented in this study suggest that the acidification of paddy soil severely interfered in the photosynthesis of diazotrophic cyanobacteria and induced the production of ROS, which in turn resulted in oxidative damage on diazotrophic cyanobacteria and a decrease in cell vitality.

## 1. Introduction

Nitrogen is the main nutrient element for crops. The application of N chemical fertilizer greatly improved global food production. However, long-term excessive fertilization using N chemical fertilizer caused an acidification of agricultural soils due to the nitrification of unused ammonia in soil [1]. The released H^+^, caused by the nitrification of ammonia, could leach soil cations such as Ca^2+^, Mg^2+^, K^+^, and increase aluminum toxicity on crops [2]. Although the effects of acidification on the physicochemical characteristics of soil and its negative impacts on the growth and yield of crops were intensively studied, the information about the effect of acidification on microorganisms in agricultural soil is limited.

Diazotrophic cyanobacteria are ubiquitous in paddy fields. They play an important role in N input in paddy fields. It is estimated that the annual N input through N_2_ fixation by cyanobacteria in paddy fields reached 20–60 Kg N ha^−1^ [3]. Thus, diazotrophic cyanobacteria have great potential to reduce N chemical fertilizer loading into paddy fields, and they may be useful in preventing soil acidification. Previous studies demonstrated that the inoculation of diazotrophic cyanobacteria into paddy fields promoted the growth of rice seedlings and increased grain yield through N input, P activation, and plant growth hormones production [4,5,6,7].

Presently, the studies about effects of acidification on diazotrophic cyanobacteria mainly focus on marine ecosystems; few target agroecosystems. The reason for ocean acidification is different to that of soil acidification. Ocean acidification is caused by an increase in CO_2_ concentration in atmosphere [8], while the acidification of agricultural soils is mainly caused by the excessive fertilization of N chemical fertilizer [1]. The effects of CO_2_ and N (NH_4_^+^/NO_3_^−^) on physiological characteristics of diazotrophic cyanobacteria are different [9,10]. Thus, the responses of diazotrophic cyanobacteria to soil acidification may be different to those of ocean acidification.

Photosynthesis and respiration are the anabolism and catabolism in cyanobacteria, respectively. They are frequently used to evaluate the vitality of cyanobacteria [11,12]. The information about the effect of soil acidification on the respiration or photosynthesis of cyanobacteria presently is limited. The damage of the photosynthetic system would increase the proportion of non-photochemical de-excitation of the energy trapped by light-harvesting antenna, which is the main source of ROS in cyanobacteria [13]. Thus, photosynthetic efficiency directly relates to the production of ROS in cells of cyanobacteria [14]. An imbalance of antioxidants and oxidants would result in oxidative damage. The antioxidative system in cyanobacteria includes enzymatic antioxidants and non-enzymatic ones. Brutemark et al. (2015) reported that acidification caused by an increase in CO_2_ concentration induced oxidative damage on the lipid of *Dolichospermum* sp. (Cyanophyta) [15]. In paddy fields, the oxidative process and antioxidative defense of diazotrophic cyanobacteria to soil acidification remain unknown.

The aim of this study was to elucidate negative effects of acidification of paddy floodwater on diazotrophic cyanobacteria. We hypothesized that: (i) the acidification of paddy floodwater would interfere in electron transport in the photosystem, which would result in the production of ROS and induce oxidative stress; (ii) oxidative stress caused by the acidification of paddy floodwater would activate the antioxidative system; (iii) an imbalance within oxidative–antioxidative systems would result in oxidative damage and decrease the cell vitality of diazotrophic cyanobacteria. To test these hypotheses, the effects of acidification of simulated paddy floodwater on the photosynthesis and respiration of a diazotrophic cyanobacterium were investigated using chlorophyll fluorometer and an oxygen electrode. In addition, the oxidative–antioxidative characteristics of the diazotrophic cyanobacterium in acidified paddy floodwater were investigated.

## 2. Materials and Methods

### 2.1. Cyanobacterial Strain

The cyanobacterium *Aliinostoc* sp. YYLX235 used in this study was isolated from a paddy field in Linxiang, Hunan Province. This cyanobacterium was cultured in BG11 medium [16]. The temperature of the culture chamber was 25 ± 1 °C. The illumination intensity was 30 μmol photons m^−2^ s^−1^ with an illumination/darkness cycle of 12 h:12 h.

### 2.2. Simulation of Paddy Floodwater

Acidified soil, caused by excessive fertilization, was collected from a paddy field located in Liuyang, Hunan Province (28°22′19″ N, 113°52′00″ E). The soil pH was 5.4. Air-dried soil was ground and passed through a sieve (20 mesh). The sieved soil was mixed with deionized water at a ratio of 10:9 (*W*:*W*), with a flooding depth of 1 cm, and then incubated at 25 °C in darkness for 15 d. Floodwater in the soil–water mixture was obtained by centrifugation at 12,000× *g* for 10 min. Floodwater was sterilized by filtration using a sterilized 0.22 μm cellulose acetate membrane. The physicochemical properties of floodwater were as follows: dissolved Al^3+^, 0.013 mg L^−1^; chemical oxygen demand, 708.9 mg L^−1^, total phosphorus concentration, 15.1 mg L^−1^; NH_4_^+^-N, 5.45 mg L^−1^, NO_3_^−^-N, 2.06 mg L^−1^, total N, 36.5 mg L^−1^.

### 2.3. Experimental Design

Experiments were carried out in 250 mL Erlenmeyer flasks, containing 91 mL floodwater, 5 mL sodium citrate buffer, and 4 mL exponential phase of YYLX235. The pH of floodwater was adjusted to 4.0, 5.0, 6.0, and 7.0 (Control) by sodium citrate buffer. The final citrate concentration was 40 mM L^−1^. Prior to this study, we observed that 40 mM L^−1^ sodium citrate showed no obvious effects on the growth and photosynthesis of YYLX235. Cells of the exponential phase of YYLX235 were collected by centrifugation at 9000× *g* for 7 min and re-suspended into pH-adjusted floodwater. The initial cell population density was about 1.36 × 10^6^ cells mL^−1^. All treatments were incubated in a culture chamber under the conditions mentioned in Section 2.1. Samples were taken on the 24th h.

### 2.4. Determination of Chlorophyll Fluorescence Transients (CFT)

The CFT of YYLX235 was determined by a fluorometer (AquaPen AP-C 100, Photon Systems Instruments Co., Brno, Czech Republic) after incubation in darkness for 15 min. The actinic light was 3000 μmol photons m^−2^ s^−1^. The parameters, PI_abs_, ϕE_0_, ϕP_0_, ψ_0_, and ϕD_0_, originated from CFT were calculated using the formulas listed in Table S1 in the Supplementary Materials.

### 2.5. Determination of Respiration and Photosynthetic Oxygen Evolution

The respiration rate and oxygen evolution rate of YYLX235 were determined using an oxygen electrode (YZQ-201A, Yi Zong Qi Technology Co., Beijing, China). Cultures of YYLX235 were loaded into oxygen electrode chamber. Variations of oxygen concentration in the oxygen electrode chamber was measured at a chamber temperature of 25 °C under darkness (Respiration) or 800 μmol photons m^−2^ s^−1^ (Oxygen evolution).

### 2.6. Dinitrogen–Fixation Rate Determination

In order to induce the formation of heterocyte, the exponential phase of YYLX235 was collected by centrifugation at 9000× *g* for 7 min and re-suspended into fresh N-free BG11 medium [16]; then, it was incubated under the condition mentioned in Section 2.1. After 15 d of incubation in BG110 medium, cells of YYLX235 were collected by centrifugation and used as inoculant to set up the experiment as described in Section 2.3. Intact filaments of YYLX235 were used to determine the N_2_ fixation rate by an acetylene reduction method [9].

### 2.7. Intracellular pH Determination

The intracellular pH of YYLX235 was determined using a pH detection kit (Molecular probes, Life Technologies Inc., Carlsbad, CA, USA) based on pH-sensitive fluorescent probes which can permeate cell membranes. The procedure of pH determination was performed according to the manufacturer’s instructions.

### 2.8. Extraction of Enzymatic and Non-Enzymatic Antioxidants

Cells of YYLX235 were collected by centrifugation and re-suspended into phosphate buffer (50 mM, pH 6.8). Algal cells were homogenized by grinding together with liquid nitrogen and then centrifuged at 12,000× *g* for 15 min at 4 °C. The enzymatic and non-enzymatic antioxidants were in the supernatant. The content of total soluble protein in the supernatant was determined by a Coomassie Brilliant Blue staining method [17].

### 2.9. Activities of Enzymatic Antioxidant

Superoxide dismutase (SOD) was determined by an NBT photochemical reduction method [18]. The activity of catalase (CAT) was assayed by its capacity of decomposing H_2_O_2_ according to the method described by Upahdyaya et al. (1985) [19]. The activity of ascorbate peroxidase (APX) was assayed based on its ability to conduct a catalyzing redox reaction between ascorbate and H_2_O_2_; determination of the activity of this enzyme was performed according to the method described by Nakano and Asada (1981) [20]. Glutathione reductase (GR), glutathione-s-transferase (GST), and peroxidase (POD) were determined using the kits produced by the Nanjing Jiancheng Bioengineering Institute (Nanjing, China).

### 2.10. Determination of Non-Enzymatic Antioxidants

Ascorbic acid (AsA) can reduce ferric ion to ferrous ion, and then, the reduced ferrous ion reacts with 2,2′-bipyridine to produce red chelates, which could be determined spectrophotometrically at 525 nm. Detailed procedures for determination of the AsA were performed according the method described by Chen et al. (2006) [21]. Reduced glutathione (GSH) was determined using a 5,5′-dithiobis-(2-nitrobenzoic acid) method (Chen et al., 2006) [21]. The carotenoids in the cells of YYLX235 were extracted using 80% acetone and determined spectrophotometrically at 470, 645, and 663 nm [22].

### 2.11. Determinations of ROS and Thiobarbituric Acid Reactive Substances (TBARS)

The content of ROS in algal cells was determined by a dichlorofluorescein diethyl ester staining method [23]. Malondialdehude (MDA) is a peroxidation product of lipid. MDA can react with thiobarbituric acid (TBA) to form a red–brown compound, which could be determined spectrophotometrically at 532 nm [24].

### 2.12. Statistic Analyses

The data in this study were statistically analyzed by one-way ANOVA (LSD) using SPSS (V. 16.0, IBM, Armonk, NY, USA). Before ANOVA analysis, the equalities of error variance were checked by Levene’s test. The data without homogeneity of variance were transformed using square root or natural logarithm until they met the requirement of ANOVA analysis. Difference was regarded as significant at *p* < 0.05.

## 3. Results

### 3.1. Respiration Rate and Oxygen Evolution Rate

**Figure 1:**
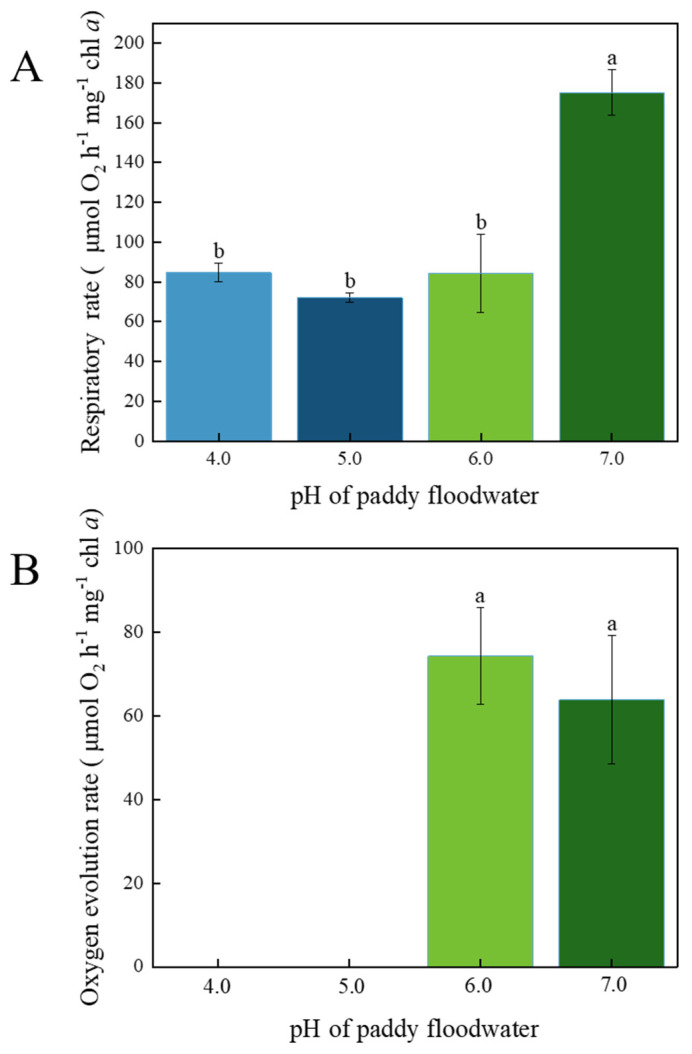
Effects of paddy floodwater acidification on respiration (**A**) and photosynthetic oxygen evolution (**B**) of *Aliinostoc* sp. YYLX235. Different letters indicate statistically significant at *p* < 0.05. (One-way ANOVA, LSD).

As shown in Figure 1, the respiration rates of YYLX235 in the acidified groups (pH 4.0–6.0) were significantly lower than that of the neutral control (pH 7.0); it was 48.4%, 41.2%, and 48.2% of the neutral control at the pH of 4.0, 5.0, and 6.0 respectively. Compared with the neutral control, photosynthetic oxygen evolution was not significantly influenced by slight acidification (pH 6.0), but it was significantly depressed in the treatments of pH 4.0 and 5.0, which fell below the resolution limit of the oxygen electrode (10 μg L^−1^).

### 3.2. Characteristics of CFT

**Figure 2:**
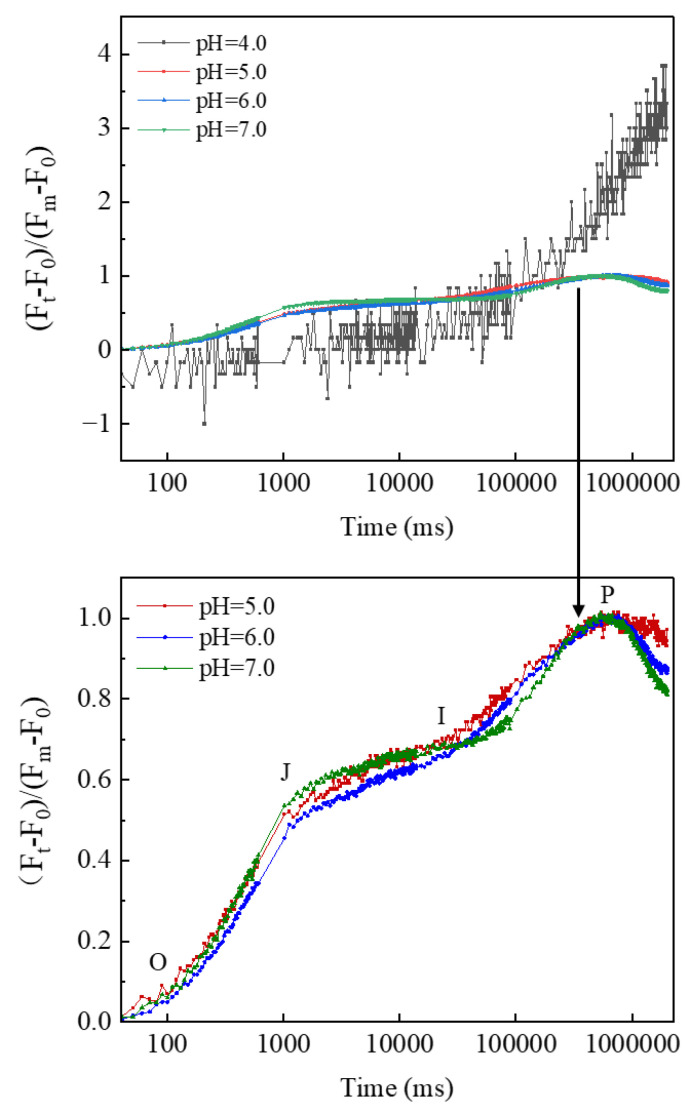
Effects of paddy floodwater acidification on the chlorophyll fluorescence transients of *Aliinostoc* sp. YYLX235.

**Figure 3:**
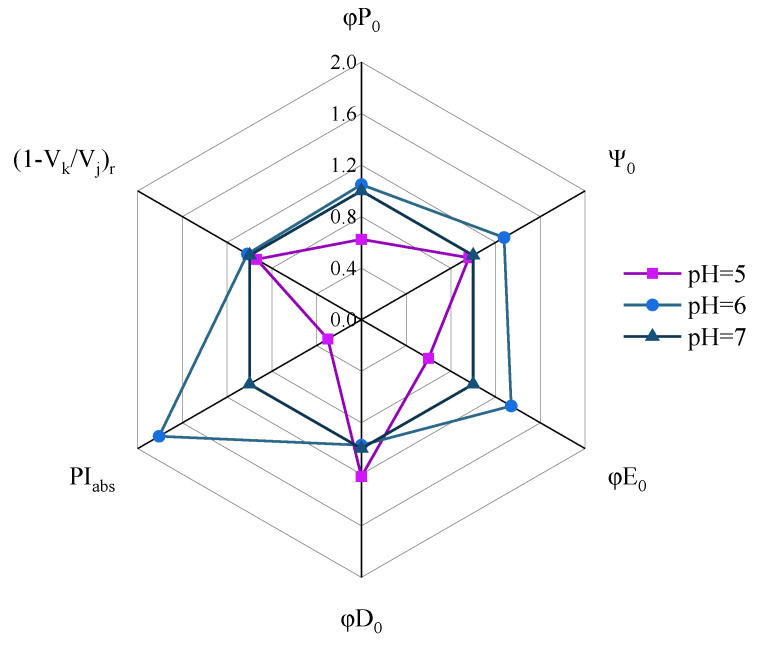
The parameters of PI_abs_, ϕE_0_, ϕP_0_, ψ_0_, ϕD_0_, and (1 − V_k_/V_j_)_r_ deviated from chlorophyll fluorescence transients. The values of each parameter were normalized using that of neutral control (pH 7.0).

Figure 2 shows the CFT of YYLX235 under different pH. Typical phases of J, I, and P were observed in the CFT of the treatments of pH 7.0, 6.0, and 5.0. However, when the pH fell to 4.0, no typical J-I-P phases were observed in the CFT. Thus, the CFT in the treatment of pH 4.0 received no further energy flux analysis. Six indexes reflecting the status of energy flux of PS II, viz. PI_abs_, ϕE_0_, ϕP_0_, ψ_0_, ϕD_0_, and (1 − V_k_/V_j_)_r_, were calculated from CFT. The meanings of these indexes are listed in Table S1. Compared with the neutral control (pH 7.0), the performance index PI_abs_ was improved at pH 6.0 but severely depressed at pH 5.0 (Figure 3). The variation of ϕE_0_ was similar to that of PI_abs_. The index ϕP_0_ was not influenced at slight acidification (pH 6.0) but significantly depressed at pH 5.0. The index ψ_0_ in the treatment of pH 5.0 showed no obvious difference to that of the neutral control, but it was higher than the neutral control in the treatment of pH 6.0. Slight acidification (pH 6.0) did not influence the value of ϕD_0_, but this index in the treatment of pH 5.0 was higher than that of the neutral control. Acidification showed no obvious effect on the index (1 − V_k_/V_j_)_r_.

### 3.3. Intracellular pH and N_2_-Fixation Rate

**Figure 4:**
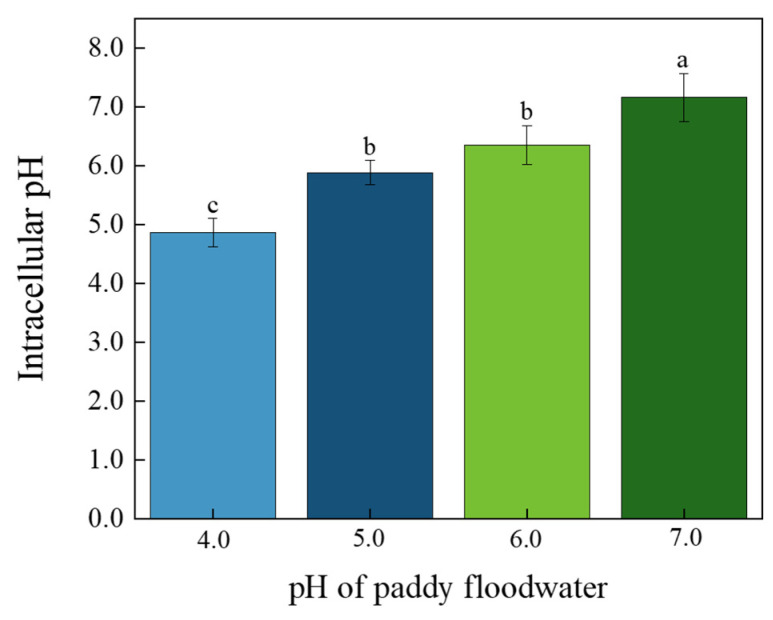
Effects of paddy floodwater acidification on intracellular pH of *Aliinostoc* sp. YYLX235. Different letters indicate statistically significant at *p* < 0.05 (one-way ANOVA, LSD).

The results of intracellular pH determination showed that it was significantly decreased by the acidification of paddy floodwater (Figure 4). Intracellular pH was 4.8, 5.8, 6.3, and 7.1, corresponding to the treatment of floodwater pH of 4.0, 5.0, 6.0, and 7.0, respectively.

**Figure 5:**
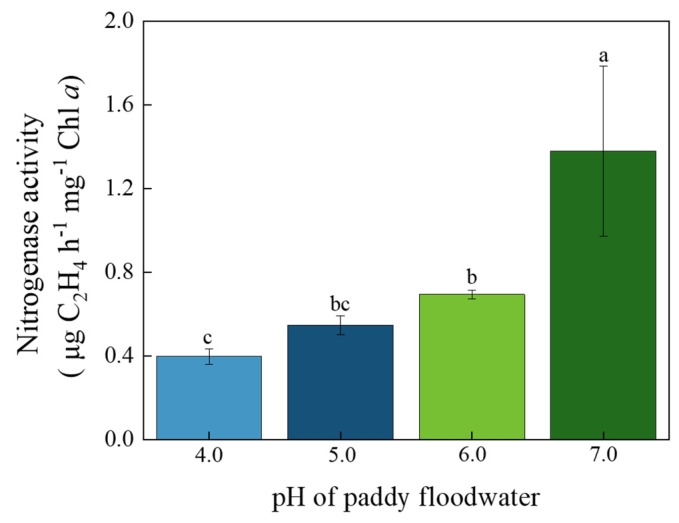
Effects of paddy floodwater acidification on N_2_ fixation rate of *Aliinostoc* sp. YYLX235. Different letters indicate statistically significant at *p* < 0.05 (one-way ANOVA, LSD).

As shown in Figure 5, the acidification of paddy floodwater had significantly negative effects on the N_2_ fixation rate of YYLX235. Compared with the neutral control, the N_2_ fixation rate in the treatment of pH 6.0, 5.0, and 4.0 decreased 49.6%, 60.4%, and 71.2%, respectively.

### 3.4. Activities of Antioxidative Enzymes

**Figure 6:**
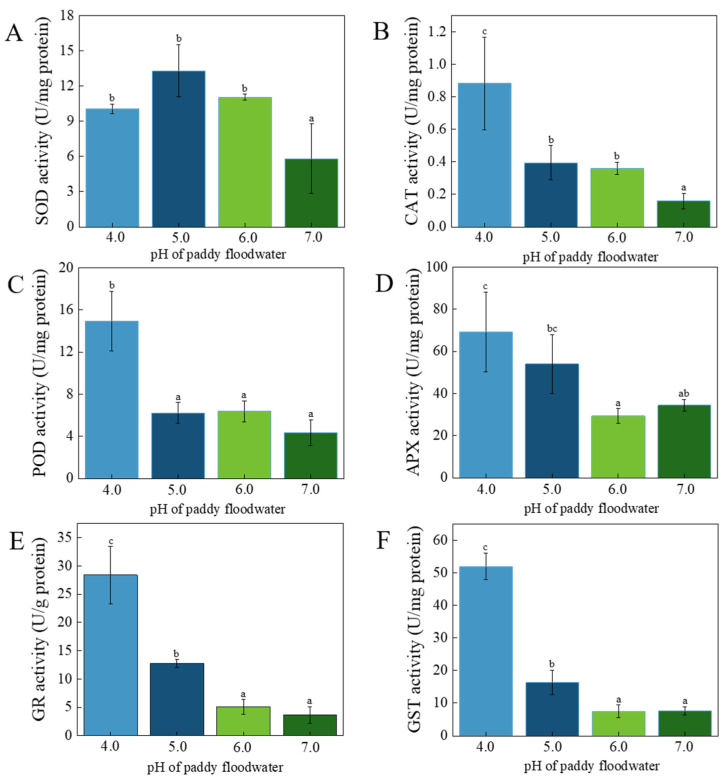
Effects of paddy floodwater acidification on activities of SOD (**A**), CAT (**B**), POD (**C**), APX (**D**), GR (**E**), and GST (**F**) in the cells of *Aliinostoc* sp. YYLX235. Different letters indicate statistically significant at *p* < 0.05 (one-way ANOVA, LSD).

Six antioxidative enzymes, viz. SOD, CAT, POD, APX, GR, and GST, were determined in this study. The activity of SOD in each acidified treatment was significantly higher than that of the neutral control (Figure 6A); it was 173%, 229%, and 190% of the neutral control for the treatment of pH 4.0, 5.0, and 6.0, respectively. The activities of CAT in the acidified treatments were also significantly higher than that of the neutral control (Figure 6B), accounting for 552%, 247%, and 225% of the neutral control for the treatment of pH 4.0, 5.0, and 6.0, respectively. The activity of POD and APX exhibited a similar trend in response to acidification (Figure 6C,D); at pH of 5.0 and 6.0, the two antioxidative enzymes were not influenced relative to neutral control, but they were significantly increased at pH 4.0. The activity of POD and APX in the pH 4.0 treatment was 342% and 218% of the neutral control, respectively. The variation of GR activity was similar to that of GST (Figure 6E,F). Compared with the neutral control, activities of GR and GST were not obviously influenced by slight acidification (pH 6.0), but they were significantly increased at pH of 4.0 and 5.0. The activity of GR in the treatments of pH 4.0 and 5.0 were 776% and 348% of the neutral control, respectively. The activity of GST in the treatment of pH 5.0 was 214% of the neutral control, and it was 680% of the neutral control in the treatment of pH 4.0.

### 3.5. Contents of Non-Enzymatic Antioxidants

**Figure 7:**
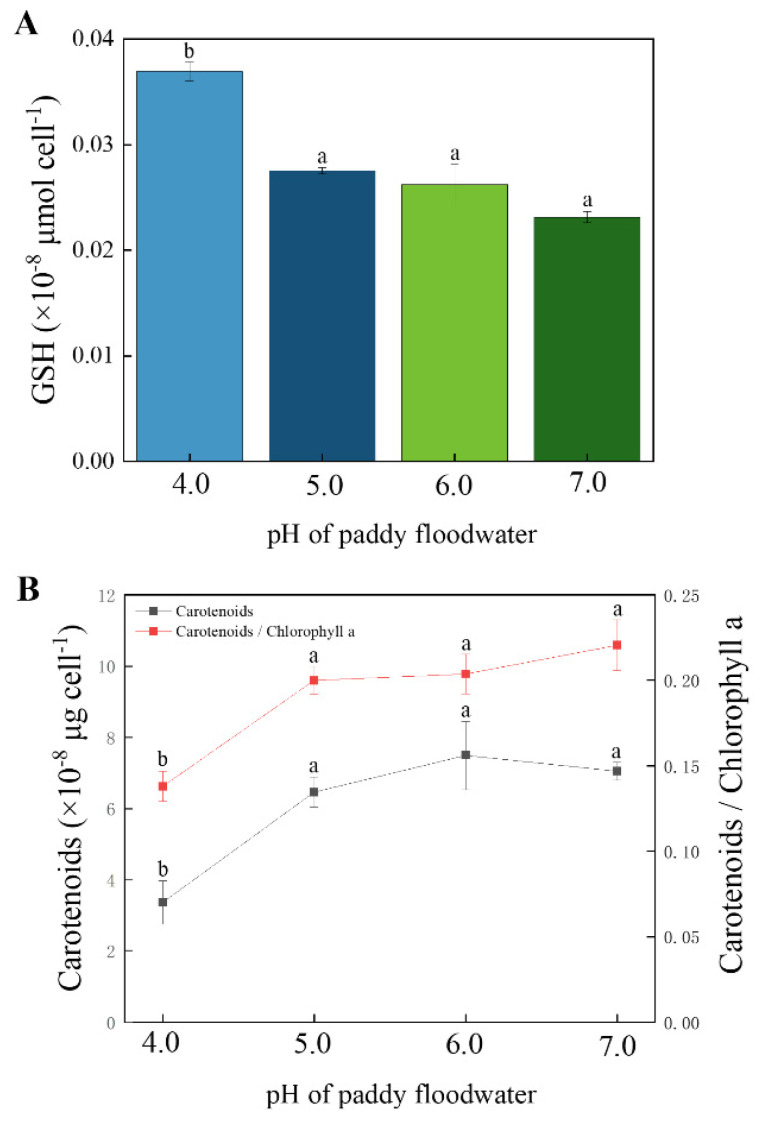
Effects of paddy floodwater acidification on levels of non-enzymatic antioxidants reduced GSH (**A**) and carotenoids (**B**) in the cells of *Aliinostoc* sp. YYLX235. Different letters indicate statistically significant at *p* < 0.05 (one-way ANOVA, LSD).

As shown in Figure 7, acidified treatments (pH 5.0 and pH 6.0) did not affect the content of GSH in cells of YYLX235, but it was significantly higher than that of the neutral control in the treatment of pH 4.0, accounting for 159% of the control. For the content of carotenoids, it was not significantly influenced at pH of 5.0–6.0 but significantly lower than the neutral control at a pH of 4.0. The ratio of carotenoids to chlorophyll *a* exhibited a similar variational trend to that of carotenoids content. The acidification of paddy floodwater had no significant effect on the ASA content in cells of YYLX235.

### 3.6. Contents of ROS and MDA

**Figure 8:**
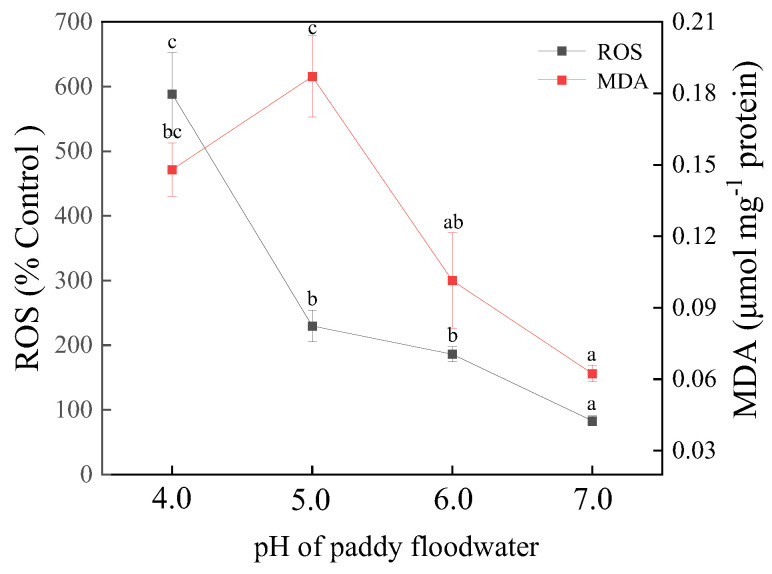
Effect of paddy floodwater acidification on the contents of cellular ROS and MDA of *Aliinostoc* sp. YYLX235. Different letters indicate statistically significant at *p* < 0.05 (one-way ANOVA, LSD).

The effects of floodwater acidification on the contents of intracellular ROS and MDA are shown in Figure 8. Acidified treatment (pH 5.0–6.0) induced an increase in the ROS content in cells of YYLX235 when compared with the neutral control, and it further boosted when the pH fell to 4.0. The ROS content in cells of YYLX235 was 223.9%, 276.6%, and 708.2% of the neutral control for the treatment of pH 6.0, 5.0, and 4.0, respectively. Slight acidification (pH 6.0) had no significant effects on the content of MDA, but it was far higher than that of the neutral control when the pH of floodwater decreased to 5.0 or 4.0.

## 4. Discussion

Activities of respiration and photosynthesis could reflect the vitality of microorganisms [25,26]. The respiration rate of YYLX235 in the acidified treatments (pH 6.0–4.0) was far lower than the neutral control, indicating that the acidification of paddy floodwater decreased cell vitality of this diazotrophic cyanobacterium. Photosynthetic oxygen evolution indicates the function of the photosynthetic system [27]. As shown in Figure 1, the oxygen evolution was severely inhibited at pH of 5.0 or 4.0, suggesting that paddy floodwater acidification interfered in the photosynthesis of YYLX235. The OJIP test of CFT reflects the structure and function of PSII [28]. There was no typical J, I, and P phases in the treatment of pH 4.0, meaning that PS II was severely damaged when the pH fell to 4.0. The PI_abs_ is a general index reflecting the performance of PSII [29]. As indicated in Figure 3, PI_abs_ was severely inhibited by the acidification of paddy floodwater. The indexes ϕP_0_ and ψ_0_ indicate the electron flux rate of the donating side and accepting side of the PSII reaction center, respectively [28,30]. The decrease in ϕP_0_ means that paddy floodwater acidification resulted in the damage of the electron-donating side of the PSII reaction center. Interfering in electron flux in photosystem II in other photosynthetic organisms, such as *Ulva prolifera* (a green algae) and leaves of tomato, were also observed when they were under the stress of simulated acid rain [31,32].

Oxygen could deactivate nitrogenase [33]. For filamentous cyanobacteria in Nostocales, they differentiate a special cell named heterocyst, which could separate nitrogenase from O_2_ [34]. The PS II in heterocyst is inactivated or lacks water-splitting capabilities, but the electron transport in PS I and bioenergetics production are still working [35]. The percentage of heterocyst in the filament of YYLX235 was about 3.8%. Thus, the photosynthesis efficiency determined in this study mainly reflected the status of vegetative cells rather than heterocysts. As presented in Figure 1, Figure 3 and Figure 4, the inhibitory rate of paddy floodwater acidification on the photosynthesis of YYLX235 (mainly vegetative cells) was more severe than that on nitrogenase activity. One possible reason for this phenomenon is the protection of heterocyst on nitrogenase due to its special structure.

Dinitrogen fixation is a high energy-consuming process. The fixation of one molecule N_2_ requires 16 molecules of ATP [36]. The bioenergetics for N_2_ fixation is provided by the photosynthesis of vegetative cells or heterocysts (PS I) [35,37]. The decrease in photosynthetic efficiency in vegetative cells, induced by the acidification of paddy floodwater, may decrease ATP production in cells of YYLX235, which may be a reason for the low N_2_ fixation rate of this cyanobacterium in acidified paddy floodwater. Previous study indicated that sea water acidification, caused by increase in CO_2_ concentration, decreased the intracellular pH of *Trichodesmium erythraeum* [9]. In order to maintain intracellular pH homeostasis, more electrons were allocated to H^+^ instead of N_2_ fixation [9]. In this study, the intracellular pH of YYLX235 treated with acidified paddy floodwater was lower than that of the neutral control. Maybe as proposed by Hong et al. (2017) [9], the lower proportion of electrons allocated to N_2_ fixation was another reason for the low N_2_ fixation rate of this cyanobacterium in acidified paddy floodwater.

ROS are by-products of aerobic metabolism. In cyanobacteria, singlet oxygen (^1^O_2_) is produced by PSII, while the superoxide anion (O_2_^−^) is produced by photosystem I [14]. In this study, the electron transport efficiency of PS II of YYLX235 decreased along with the decrease in floodwater pH. It means that under an acidified environment, more excited energy trapped by a light-harvesting antenna complex may be transformed into ^1^O_2_. SOD is the unique antioxidant enzyme which dismutates O_2_^−^ into H_2_O_2_ and O_2_. Although the electron transport efficiency of PS I was not checked in this study, the increase in activity of SOD in the acidified treatments suggests that the acidification of paddy floodwater (pH 4.0–6.0) increased the production of O_2_^−^ by PSI of YYLX235.

For the six antioxidative enzymes determined in this study, in the treatment of pH 6.0, only the activities of SOD and CAT were significantly up-regulated relative to the neutral control, suggesting that SOD and CAT were sensitive antioxidative enzymes in cells of YYLX235. Hydrogen peroxide could be hydrolyzed by CAT, POD, and APX [14]. As shown in Figure 6, the activity of CAT significantly increased in response to the acidification of floodwater even at a pH of 6.0, while the activities of POD and APX were not influenced at pH of 6.0 and 5.0. Thus, we deduce that CAT was the main H_2_O_2_ scavenger in the cells of YYLX235 in response to the acidification of paddy floodwater.

GST is a kind of important enzyme in the detoxification of ROS and some toxins in mammalian cells and some prokaryotes including cyanobacteria [38]. Recently, GST was evidenced to be essential to the growth of *Synechocystis* PCC 6803 (Cyanobacteria) under illumination conditions [39]. In this study, the activity of GST was more sensitive to acidification (increasing as pH of paddy floodwater decreased) than those of POD and APX, suggesting that GST in the cells of YYLX235 may play an important role in coping with oxidative stress induced by the acidification of paddy floodwater. Generally, the structural stability of GST is restricted to a narrow pH range around neutral environment. However, a study from Pandey et al. (2017) demonstrated that the GST from *Synechocystis* PCC 6803 could maintain its activity over a broad pH range from 2.0 to 11.0 [40]. The results of intracellular pH determination evidenced that YYLX235 maintained GST activity at a pH of 4.8. Considering that YYLX235 was isolated from an acidified paddy soil, we deduce that evolutionary selection in acidic environments extended the pH range of GST stability in YYLX235.

The antioxidative system in cyanobacteria includes enzymatic antioxidants and non-enzymatic antioxidants [41]. Carotenoids, AsA, and GSH are the main non-enzymatic antioxidants in cyanobacteria. The responses of non-enzymatic antioxidants in cyanobacteria are species dependent and stress-type dependent [41,42,43]. Lin et al. (2019) reported that, after being induced by dissolved organic matter, an increase in intracellular ROS in *Microcystis aeruginosa* was accompanied by an increase in carotenoids content and the ratio of carotenoids to chlorophyll *a* [44]. However, this phenomenon was not observed in this study. Either the carotenoids content or the ratio of carotenoids to chlorophyll *a* was decreased by acidification (pH 4.0), suggesting that carotenoids may not be the main non-enzymatic antioxidants in the YYLX235 when it was under the stress of acidification. For the three non-enzymatic antioxidants, viz. carotenoids, AsA, and GSH in this study, only reduced GSH was up-regulated in the acidified treatments, indicating that reduced GSH was the main non-enzymatic antioxidant in the cells of YYLX235 in response to acidification of paddy floodwater.

Antioxidants and oxidants in a cell are usually in balance. Thus, although ROS continue to be produced during photosynthesis and other aerobic metabolisms in cyanobacteria, no oxidative damage happens when it is under normal condition. When a cell faces weak intensity of oxidative stress, levels of antioxidants may increase to mitigate oxidative damage [45]. In this study, the content of intracellular ROS in the treatment of pH 6.0 was higher than that of the neutral control, and the levels of antioxidants were also increased, indicating that a slight acidification of paddy floodwater (pH 6.0) induced oxidative stress. However, the results of lipid oxidative damage showed that at a pH of 6.0, lipid oxidative damage was not aggravated relative to the neutral control, suggesting that the antioxidative system of YYLX235 could eliminate some sources of oxidative stresses induced by the slight acidification of paddy floodwater. The parameters originated from CFT indicated that the electron transport efficiency of photosystem II in the treatment of pH 6.0 was higher than the neutral control. Debnath et al. (2018) reported that increases of antioxidants induced by melatonin mitigated the damage of simulated acid rain on electron flux in PS II of tomato leaves [32]. Thus, we deduce that protection by the increase in antioxidants may partially contribute to this phenomenon. The hormesis of a photosystem under an acidified environment may be another reason responsible for this phenomenon [46]. When the pH of paddy floodwater fell to 5.0–4.0, even though the activities of enzymatic antioxidants and the content of reduced GSH increased, lipid oxidative damage in these acidified treatments was more severe than that of the neutral control, suggesting that antioxidants were not able to keep balance with oxidants when the pH of floodwater fell to the level of ≤5.0.

## 5. Conclusions

The excessive use of N chemical fertilizer often leads to the acidification of paddy soil. The effects of paddy floodwater acidification on the physiological characteristics of *Aliinostoc* sp. YYLX235 were investigated in this study. The acidification of paddy floodwater decreased activities of respiration, photosynthesis, and N_2_ fixation of YYLX235. The levels of antioxidants in the cells of YYLX235 were increased in response to the acidification of paddy floodwater, but they could not eliminate oxidative stress when the pH of paddy floodwater fell to 5.0 and 4.0, and lipid oxidative damage happened. The results presented in this study suggest that the acidification of paddy floodwater induced oxidative stress on diazotrophic cyanobacteria and decreased cell vitality.

## Data Availability

The data presented in this study are available on request from the corresponding author.

## Supplementary Materials

The following supporting information can be downloaded at: https://www.mdpi.com/article/10.3390/ijerph192215070/s1, Table S1: The formulae and the illustration of the parameters derived from OJIP chlorophyll fluorescence transients.
