# Supplementary material for: Physiological Responses of a Diazotrophic Cyanobacterium to Acidification of Paddy Floodwater: N2 Fixation, Photosynthesis, and Oxidative–Antioxidative Characteristics
Source: Int J Environ Res Public Health. 2022 Nov 16;19(22):15070. doi: 10.3390/ijerph192215070 (PMC9690652; doi:10.3390/ijerph192215070)
Supplement: Supplementary file 1 [file ijerph-19-15070-s001.zip › ijerph-1992031-supplementary.pdf]

Table S1. The formulae and the illustration of the parameters derived from OJIP chlorophyll fluorescence transients.

**Formulae**	**Illustration**
$\varphi P_0 = 1-(F_0/F_M)$	Maximum quantum yield of primary photochemistry
$\psi_0 = 1-V_J$	Probability that a trapped exciton moves an electron further than Q_A^-
$\varphi E_0 = (1-F_0/F_M)(1-V_J)$	Probability that an absorbed photon moves an electron further than Q_A^-
$\varphi D_0 = F_0/F_M$	Maximum quantum yield of non photochemical deexcitation
$(1-V_K/V_J)_r = [(1-V_K/V_J)_{\text{treatment}}]/[(1-V_K/V_J)_{\text{Control}}]$	State of oxygen evolution complex
$PI_{abs} = [RC/ABS][\varphi P_0/(1-\varphi P_0)][\psi_0/(1-\psi_0)]$	General performance index of photosynthesis
